# A case of endovascular management to gain control of a lower gastrointestinal haemorrhage caused by appendiceal artery bleeding

**DOI:** 10.1093/jscr/rjab204

**Published:** 2021-06-10

**Authors:** Eleanor J Smith, Charles Coventry, Jeremy Taylor, Henry De’ath, Ali Haque

**Affiliations:** Surgical Department, Harris Suite, University Hospital Lewisham, London SE13 6LH, UK; Frimley Park Hospital, Camberley GU16 7UJ, UK; Frimley Park Hospital, Camberley GU16 7UJ, UK; Frimley Park Hospital, Camberley GU16 7UJ, UK; Frimley Park Hospital, Camberley GU16 7UJ, UK

## Abstract

Bleeding from the appendix is a rare cause of lower gastrointestinal haemorrhage. Previous publications have noted diagnosis via colonoscopy or computed tomography angiogram and treatment via surgical or endoscopy. We report a case of large volume per rectal bleeding from the appendix, with diagnosis and treatment via angiography and coil insertion, which is the first of its kind reported in the literature.

## INTRODUCTION

Lower Gastrointestinal bleeding (LGIB) accounts for 30% of surgical referrals in the UK [1]. After initial resuscitation, identifying the source of the bleeding is a clinical priority, which can be done via endoscopy to allow direct visualization of lumen, computed tomography (CT) scan of the abdomen and pelvis, or dedicated CT angiography (CTA) [2]. If an active bleed is identified on CTA, this enables the option of embolization, however this is used in <1% of cases [2]. Appendix as the source of bleeding in uncommon [3]. The object of this publication is to present an unusual case of LGIB and the use of embolization to haemostasis, and to review previous published literature of similar cases and their methods of treatment.

## CASE REPORT

A 67-year-old man presented to Accident and Emergency with a 1-day history large volumes of bright red per rectal bleeding. He denied any previous episodes. The patient had no previous surgical history, no history of gastrointestinal disturbance, and no change in bowel habit, weight loss, lethargy, abdominal pain or any infective symptoms. He had a background of insulin-dependent diabetes, hypertension, ischaemic heart disease and asthma. At time of initial surgical assessment, he had a systolic blood pressure of 103 mm Hg and a heart rate of 93 bpm. Abdominal examination did not identify any specific abnormalities and per rectal examination demonstrated fresh blood on the glove. The initial haemoglobin level was 158 mg/dl.

**Figure 1:**
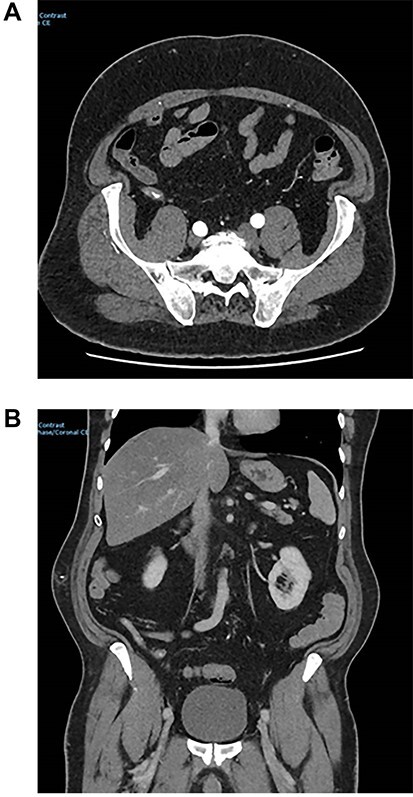
CT images of arterial appendix blush.

During initial surgical assessment, the patient identified that he was a practicing Jehovah’s Witness and would not accept blood products, even if that would result in a poor outcome, including death. The patient was therefore resuscitated intravenously with crystalloids as per his expressed wishes and underwent a CT angiogram to investigate the cause of the bleeding. CT scan demonstrated active bleeding in the caecum/appendix with an obvious blush noted (Fig. 1).

Despite an 18-hour period of conservative management, the patient continued to active bleed. Following discussion with the consultant surgeon, the consultant interventional radiologist and the patient, it was decided to proceed to a mesenteric angiogram to specifically identify and treat the area of bleeding. The patient’s beliefs as a Jehovah’s witness and his decision to refuse blood products did factor in this decision. Although he was stable, it was felt that it would be best to avoid a large operation, such a right hemicolectomy which may be required to control the bleeding operatively. Without the option of blood products, the morbidity and mortality from such a procedure could be much higher.

**Figure 2:**
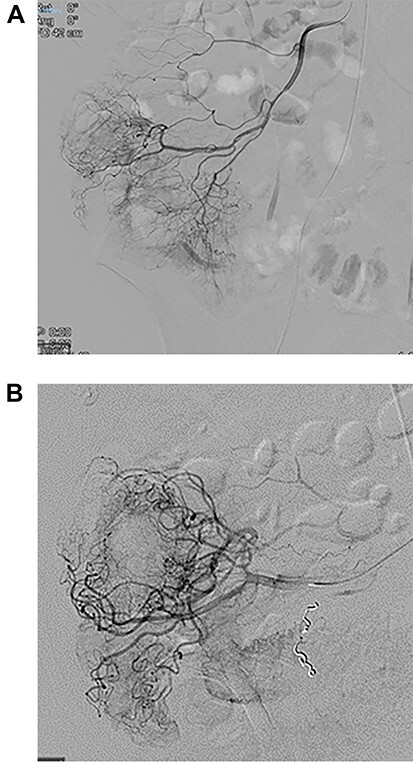
Images from angiography. (**A**) Pre-embolization, demonstrates appendiceal lumen filled with contrast. (**B**) Demonstrated coiled artery.

At angiogram, the superior mesenteric artery (SMA) was successfully cannulated, and the patient was found to have active extravasation from the appendiceal artery (Fig. 2). This was successfully controlled with a 3 mm coil.

**Figure 3:**
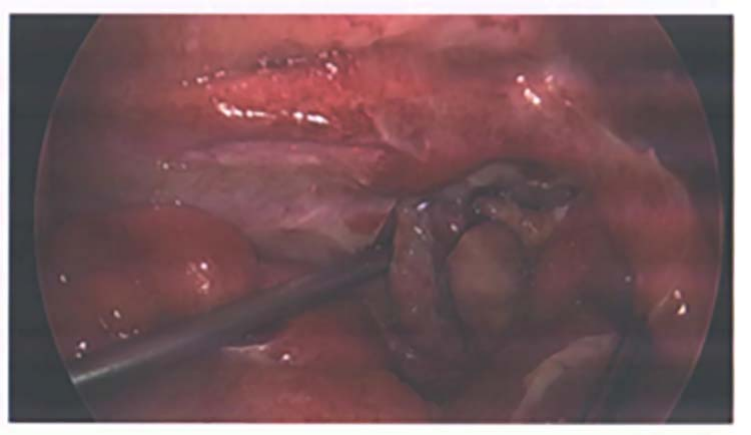
Image from laparoscopic appendicectomy.

Due to the embolization of an end artery, the high possibility of ischaemia of the appendix was a concern and the patient was closely monitored for signs of rebleeding and peritonism. He was also prophylactically placed on IV antibiotics per trust guidelines for intra-abdominal pathology. After 72 hours of conservative management, he exhibited signs of local peritonism in the right iliac fossa and a rising white cell count and the decision was made to proceed with operative management. A laparoscopic appendicecetomy was performed, and he was found to have an inflamed appendix with blue discoloration indicating ischemia (Fig. 3). No perforation or other abnormalities throughout the examined bowel were identified. Final histology demonstrated partial distal infarction, healthy viable base and no sign of neoplasm. The patient had an unremarkable recovery following his operation and was discharged after 2 days.

## DISCUSSION

The BSG guidelines published in 2019 recommended that CTA should be the first-line investigation in patients with an active LGIB [4]. The most common cause of LGIB is diverticular bleeding, followed by benign anorectal conditions [2]. Overall, the most common intervention for LGIB is a red blood cell transfusion [5]. Therefore, patients who decline blood products can present challenges for an acute surgical team [5]. The British Society of gastroenterology guidelines in 2019 defined unstable LGIB as those with a shock index of >1 and suggest that these patients should have a CTA as a first line [4]. Although our patient only had a shock index of 0.9, this was still above the normal range (0.5–0.7) and in this instance, when the option of transfusion was not available, we chose to intervene early with angiography. The patient was actively bleeding for several hours and we did not want to risk haemodynamic instability in a patient refusing blood products. Any operative intervention’s potential morbidity for this gentleman would be higher due to his decision to decline blood products.

A review of the literature confirmed that appendix as the source of a LGIB is rare, with 27 papers identified which reported such an occurrence, from a variety of different underlying pathologies. The majority of the papers reported operative management was performed as first line, however there were four cases that underwent an endoscopic intervention either colonoscopic clipping [6, 7] or barium enema at colonoscopy [8]. We did not identify any previous use of endovascular techniques to gain emergency control of a large appendiceal LGIB.

In this case we were able to demonstrate the safe use of endovascular intervention to control an appendiceal haemorrhage in a patient with additional compounding factors. By gaining control of the bleeding via angiography, prior to undergoing a routine operation in the following days, when he was less likely to become haemodynamically unstable, we were able to perform an operation with much a lower risk of morbidity and mortality.

## INFORMED CONSENT

Written informed consent was obtained from the patient for publication of this case report and accompanying images. A copy of the written consent is available for review by the Editor-in-Chief of this journal on request.
